# Assessing causal relationships between gut microbiota and abortion: evidence from two sample Mendelian randomization analysis

**DOI:** 10.3389/fendo.2024.1415730

**Published:** 2024-07-10

**Authors:** Hang Yao, Jiahao Chen, Yu Wang, Yuxin Li, Qingling Jiang

**Affiliations:** ^1^ School of Traditional Chinese Medicine, Binzhou Medical University, Yantai, China; ^2^ School of Basic Medical Sciences, Zhejiang Chinese Medical University, Hangzhou, China; ^3^ Graduate School of Jiangxi University of Traditional Chinese Medicine, Nanchang, China

**Keywords:** gut microbiota, Mendelian randomization analysis, spontaneous abortion, habitual abortion, causal relationship

## Abstract

**Background:**

While some studies have suggested a link between gut microbiota (GM) and abortion, the causal relationship remains unclear.

**Methods:**

To explore the causal relationship between GM and abortion, including spontaneous abortion (SA) and habitual abortion (HA), we performed a two-sample Mendelian randomization (MR) analysis. We used summary statistics data from MiBioGen and FinnGen for genome-wide association studies (GWAS), with GM data as the exposure variable and abortion data as the outcome variable.

**Results:**

In the absence of heterogeneity and horizontal pleiotropy, the inverse-variance weighted (IVW) method identified five genetically predicted GM genera linked to the risk of abortions. *Lactococcus* was negatively correlated with the risk of SA, whereas the *Eubacterium fissicatena* group was positively correlated with the risk of SA. Genetic predictions of *Coprococcus3* and *Odoribacter* were linked to a reduced risk of HA, while the *Eubacterium ruminantium* group was associated with an increased risk of HA.

**Conclusion:**

Our study suggests a genetic causal relationship between specific GM and two types of abortions, improving our understanding of the pathological relationship between GM and abortion.

## 1 Introduction

Abortion, commonly referred to as miscarriage, is a frequent complication in early pregnancy, usually occurring before the 20th week of gestation. According to the American Society for Reproductive Medicine (ASRM), 15-25% of pregnant women experience miscarriages, although the actual rate may be higher in reality (1). The causes of abortion are varied and complex, with chromosomal abnormalities believed to account for about 50% of cases globally (2). Despite this, the mechanisms behind abortion remain largely unknown (3). In cases of threatened abortion, medical professionals often prescribe hormones like progesterone and dydrogesterone, but their prolonged use can result in emotional disturbances and other pregnancy complications (4, 5). A 2021 report by The Lancet emphasized that the consequences of abortion extend beyond personal and family distress, affecting national health systems and societal economics (1). Therefore, it is crucial to address the negative impacts of abortion and prevent potential risk factors.

The gut microbiota (GM), the most complex microbial community in the human body, plays a significant role in health and disease (6, 7). It has been a focal point of life sciences research for decades. The GM can influence female pregnancy through mechanisms such as immunity regulation, metabolism, inflammation, and the gut-uterine axis (8–10). The balance of microbial communities within the endometrium directly affects reproductive outcomes and may be a factor in recurrent miscarriages (11). Current evidence suggests that changes in certain GM components may support healthy pregnancies, while an imbalance in GM is associated with complications, including abortion (12). Notably, butyrate produced by GM supports intestinal health and normal immune function (13, 14). A reduction in butyrate has been observed in patients with recurrent abortions, drawing researchers’ attention (15).

Mendelian randomization (MR) is an epidemiological technique that uses single nucleotide polymorphisms (SNPs) as instrumental variables (IVs) to estimate the causal effects of specific exposures on outcomes (16). This method is particularly valuable in medical research because it can minimize the influence of confounding factors, thus offering significant potential for exploring causal relationships in healthcare studies (17). To date, the relationship between the GM and abortion has been preliminarily investigated in observational studies, but the causal relationship between GM and abortion has not yet been explored (18).

Therefore, this study employs genome-wide association study (GWAS) data from the MiBioGen consortium and the FinnGen database to investigate the causal relationship between the GM and abortion through a two-sample MR analysis. We anticipate that this research will uncover potential pathogenic mechanisms of abortion and propose new strategies for improvement, thereby informing new directions in clinical treatment.

## 2 Materials and methods

### 2.1 Study overview

**Figure 1:**
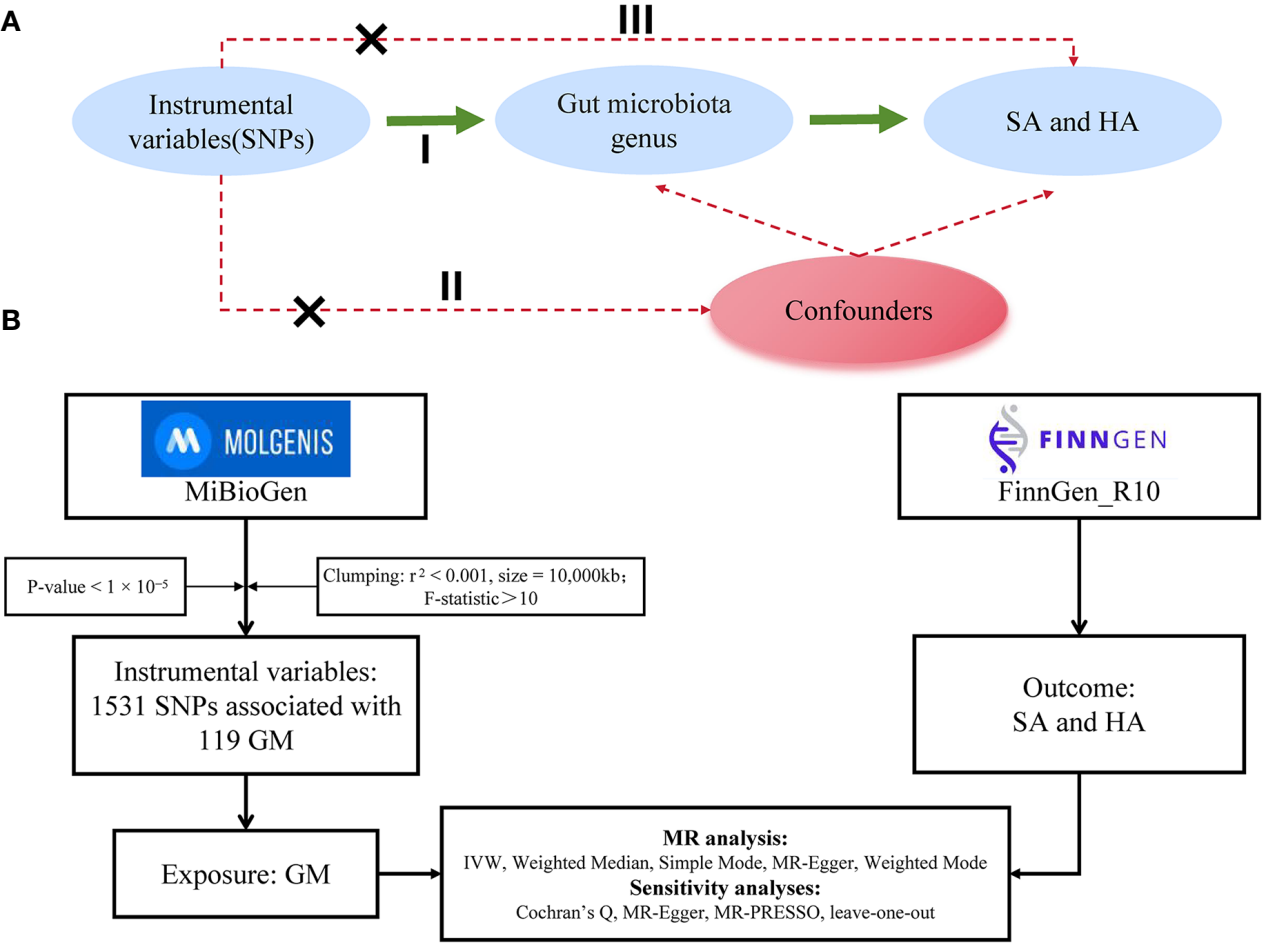
**(A)** Three assumptions of Mendelian randomization: I, correlation assumption; II, independence assumption; III, exclusionary restriction assumption. **(B)** provides a flowchart of this Mendelian randomization study. Abbreviations used include MR for Mendelian randomization, SNP for single nucleotide polymorphism, GM for gut microbiota, SA for spontaneous abortion, and HA for habitual aborter.

This study followed the framework outlined in **Figure 1**, treating each bacterial genus in the gut microbiota (GM) as an independent exposure factor and considering two types of abortion as outcome variables. The two-sample MR method was used to investigate specific microbial taxa in the GM that have a causal relationship with abortion. The MR method in this study was based on three assumptions: 1) Single-nucleotide polymorphisms (SNPs) used as instrumental variables (IVs) are associated with the GM; 2) IVs are independent of confounding factors; 3) IVs affect abortion risk solely through the GM, not through other pathways (19, 20).

### 2.2 Data sources

The MR analysis utilized two distinct genome-wide association study (GWAS) datasets. First, GM data were sourced from the MiBioGen Consortium, which conducted a large-scale population genetics study involving 18,340 individuals from 24 cohorts. The SNPs in this study were derived from human samples, initially including 14,587 SNPs (*p* < 1×10^-5^) related to the gut microbiome (21). Second, GWAS data for spontaneous abortion (SA) and habitual aborter (HA) were obtained from the FinnGen database. The SA study included 181,667 participants (18,680 cases and 162,987 controls) with a total of 21,292,180 SNPs. The HA study included 112,234 participants (651 cases and 111,583 controls) with a total of 21,266,295 SNPs. In this study, the GM was considered the exposure factor, while the two distinct types of abortion were regarded as outcome factors. SNPs were used as IVs in this study. Further details can be found in **Table 1**.

**Table 1:** Details of the GWASs included in the Mendelian Randomization.

Trait	Data Type	N_cases	N_controls	Consortium/Dataset
Gut Microbiota	Exposure	18,340		MiBioGen
Spontaneous abortion	Outcome	18,680	162,987	FinnGen_R10
Habitual aborter	Outcome	651	111,583	FinnGen_R10

### 2.3 Selection of instrumental variables

In our dataset of GM, we classified the genera at the genus level, resulting in a total of 131 genera. We excluded 12 unknown genera, leaving 119 bacterial genera for the MR analysis (22). To ensure the accuracy of the causal relationship between GM and abortion, we implemented a series of quality control procedures to select SNPs related to microbial features. First, we selected SNPs associated with the GM using a significance threshold of *p* < 1×10^-5^, ensuring a significant correlation between the selected SNPs and the GM. Second, we assessed the independence of the selected SNPs by performing a clumping process (r^2^ < 0.001, kb = 10,000) to evaluate linkage disequilibrium (LD) (23). Third, we extracted SNP information relevant to both exposure and outcome, aligning the effect alleles to ensure data accuracy. Subsequently, the F-statistic of the SNPs was employed to assess the strength and stability of the IVs in relation to the exposure factor. IVs with an F-value ≤ 10 were deemed to have a weak correlation with the exposure and were therefore excluded. The calculation formula for the F-statistic is F=β^2^ exposure/SE^2^ exposure (24).

### 2.4 Statistical methods and sensitivity analysis

This study thoroughly investigated the potential causal relationship between GM and two types of abortion using five analytical methods: Inverse Variance Weighted (IVW), Weighted Median, Simple Mode, MR-Egger, and Weighted Mode, with IVW serving as the primary method (25). To guard against false positives in multiple testing, we applied a Bonferroni correction to establish a statistically adjusted significance threshold [*p* = 4.20 × 10^-4^ (0.05/119)] (26). We assessed the heterogeneity of the results using the *p*-value from Cochran’s Q test. A *p*-value < 0.05 indicated the presence of heterogeneity, while a *p*-value > 0.05 suggested no significant heterogeneity. The reliability of the MR analysis results was validated through the intercept test using the MR-Egger method. An intercept *p*-value > 0.05 indicated the absence of horizontal pleiotropy, thereby improving the robustness of the study findings. Additionally, a leave-one-out sensitivity analysis was conducted to sequentially exclude individual SNPs and identify any SNPs with a strong influence on the MR estimates. The reliability of the results was further assessed using funnel plots and forest plots. All statistical analyses were performed using R-4.3.2 and RStudio software, utilizing the Two Sample MR package (version 0.5.7). Our rigorous methods and procedures aimed to improve the scientific quality and credibility of the research on the potential causal relationship between GM and the two types of abortion.

## 3 Results

### 3.1 Instrumental variable selection

**Figure 2:**
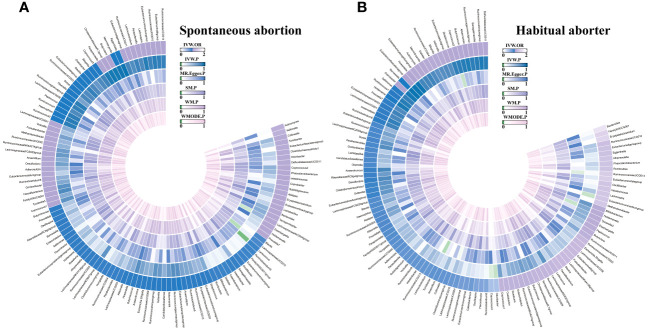
The circus plot showing the MR results of all gut microbiota. IVW, inverse-variance weighted; SM, Simple mode; WM, Weighted median; WMOED, Weighted mode; P, *p*-value; OR, odds ratio. **(A)** Spontaneous abortion; **(B)** Habitual aborter.

Based on predefined criteria, we selected 1531 SNPs as IVs for 119 GM genera. The analysis showed that the F statistics for these SNPs were greater than 10 (**Supplementary Table S1**), indicating their robustness as IVs. This suggests that there is no evidence of weak instrument bias, further confirming the reliability of the results. We presented all MR analysis results for the 119 GM genera and the risk of the two types of abortion in **Figure 2**. Additional details of the analysis results for the 119 GM genera and the two types of abortion can be found in **Supplementary Tables S2**, **S3**.

### 3.2 Effects of genetically predicted gut microbiota on two types of abortion

**Figure 3:**
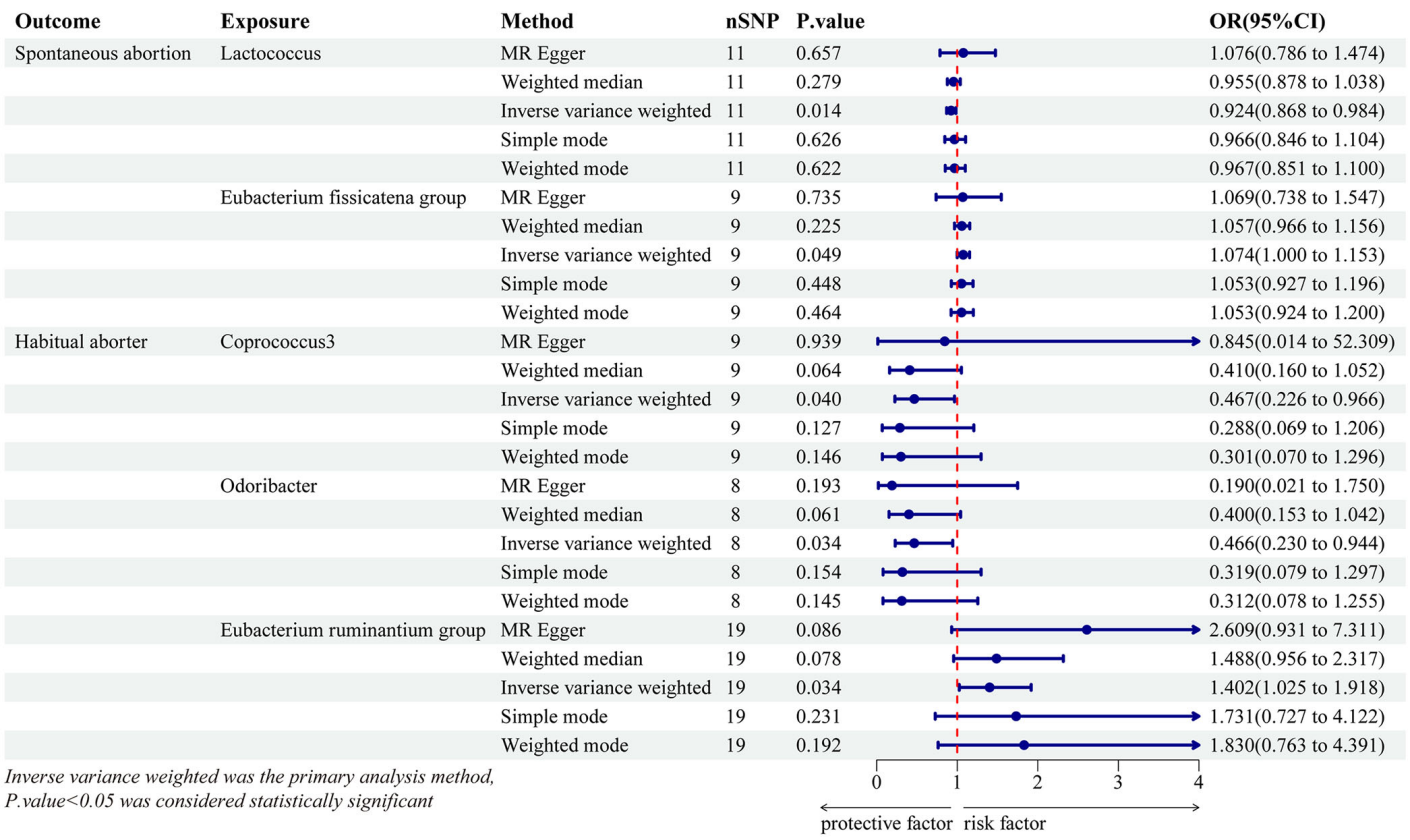
Forest plot of genetically predicted gut microbiota associated with two types of abortion by five MR methods. OR, odds ratio.

**Figure 4:**
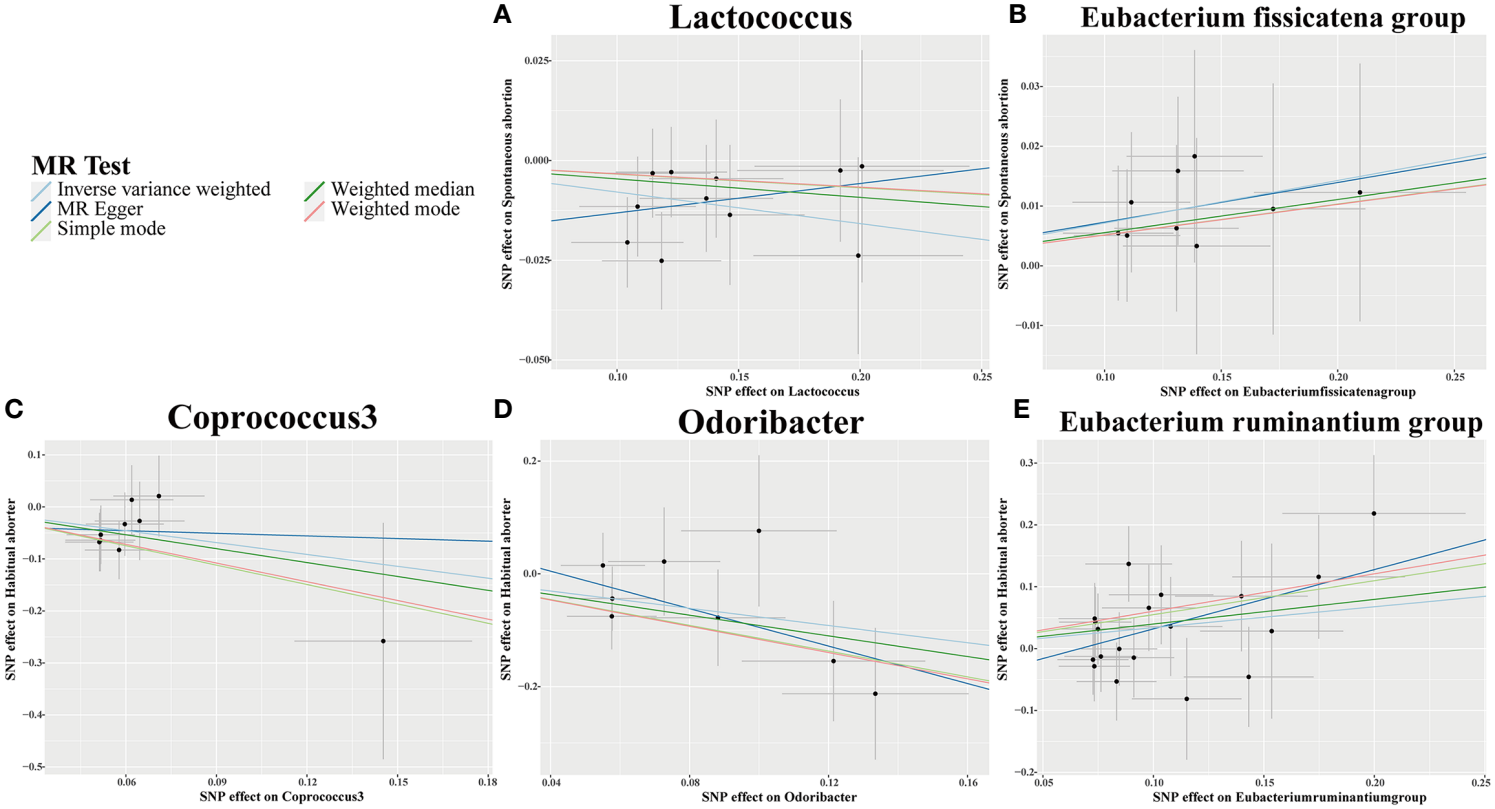
Scatter plots for causal effects of gut microbiota on 2 types of abortion risk using five MR methods. **(A, B)** Spontaneous abortion; **(C-E)** Habitual aborter.

Using IVW analysis, we identified five specific GM genera associated with the risk of abortion. *Lactococcus* (OR = 0.924, 95% CI: 0.868-0.984) exhibited a protective effect on spontaneous abortion (SA), while the *Eubacterium fissicatena* group (OR = 1.074, 95% CI: 1-1.153) was associated with an increased risk of SA. *Coprococcus3* (OR = 0.467, 95% CI: 0.226-0.966) and *Odoribacter* (OR = 0.466, 95% CI: 0.23-0.944) showed a protective effect on habitual abortion (HA), whereas the *Eubacterium ruminantium* group (OR = 1.402, 95% CI: 1.025-1.918) was associated with an increased risk of HA (**Figure 3**). A scatter plot in **Figure 4** illustrates the estimated effects of GM SNPs on abortion based on our MR analysis results.

However, despite the identified causal relationships, the observed outcomes did not meet the stringent threshold set by the Bonferroni correction and thus lost statistical significance after adjustment.

### 3.3 Sensitivity analysis

**Figure 5:**
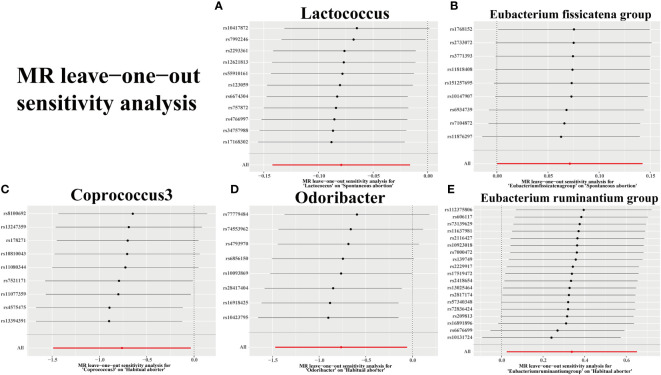
Plots for “leave-one-out” analysis for causal effect of gut microbiota on 2 types of abortion risk. **(A, B)** Spontaneous abortion; **(C-E)** Habitual aborter.

We assessed the heterogeneity of SNPs using Cochran’s Q test, as presented in **Table 2**. Additionally, we evaluated the horizontal pleiotropy of SNPs using Egger’s intercept and MR-PRESSO. The results indicated no significant heterogeneity or horizontal pleiotropy (*p* > 0.05). Further confirmation of data robustness was achieved through leave-one-out sensitivity analysis, funnel plots, and forest plots (**Figure 5**; **Supplementary Figures S1**, **S2**).

**Table 2:** Sensitivity analysis of the MR analysis results of the gut microbiota and abortions.

Outcome	Exposure	Heterogeneity		Directional pleiotropy		MR-PRESSO
		Cochran’s Q	*p*-value	Egger intercept	*p*-value	*p*-value
Spontaneous abortion	Lactococcus	3.674	0.932	-0.020	0.357	0.913
	Eubacterium fissicatena group	0.861	0.997	0.001	0.979	0.998
Habitual aborter	Coprococcus3	3.199	0.866	-0.036	0.783	0.917
	Odoribacter	3.750	0.710	0.071	0.436	0.766
	Eubacterium ruminantium group	12.712	0.755	-0.064	0.232	0.710

## 4 Discussion

This study represents the first investigation into the causal relationships between the GM and different subtypes of abortion. Abortions are categorized based on clinical presentation, such as habitual aborter, and whether they occur spontaneously, such as spontaneous abortion (4, 27–29). We sourced GM data from the MiBioGen database and data on spontaneous and habitual aborter from the FinnGen database. We conducted MR and sensitivity analyses on 119 bacterial genera and two abortion subtypes. Our research identified five bacterial genera with a causal relationship to abortion, with sensitivity analyses showing no evidence of heterogeneity or pleiotropy. These findings support our hypothesis of a causal link between GM and abortion.

The World Health Organization (WHO) defines spontaneous abortion as the natural death of an embryo or fetus before the 20th week of pregnancy without external intervention (30). Our MR analysis found two GM significantly associated with spontaneous abortion. *Lactococcus*, recognized as beneficial microbiota in healthy pregnancies, has been extensively researched for its positive effects on colitis and its ability to induce apoptosis in colorectal cancer cells (31, 32). Research by Antonio González-Sánchez indicates that *Lactococcus* is highly active in the vagina during childbirth (33). We speculate that *Lactococcus* plays a protective role during pregnancy. Additionally, our MR analysis identified the *Eubacterium fissicatena* group as a risk factor. Numerous studies have shown that the *Eubacterium fissicatena* group affects the host’s immune system and may cause pregnancy failure leading to abortion (34).

It is a common misconception that habitual abortion is simply a series of spontaneous abortions; however, this is not accurate. The American Society for Reproductive Medicine (ASRM) specifically defines Recurrent Pregnancy Loss (RPL) as experiencing two or more miscarriages before the 20th week of pregnancy. Approximately 2.5% of pregnant women experience this condition (35). The primary causes include genetic issues, uterine structural abnormalities, hormonal imbalances, and immune system problems (36), such as chromosomal abnormalities, endometritis, thyroid disorders, and Celiac disease (37–39).

In our MR study, we identified three specific microbiota associated with habitual abortion. The Eubacterium rectale group is associated with an increased risk, suggesting it may contribute to habitual miscarriages. Observational studies by Yongjie Liu have shown an increased abundance of the Eubacterium rectale group in the feces of women with habitual abortions, indicating its role in increasing this risk (18). Conversely, *Odoribacter* and *Coprococcus3* appear to have protective roles against habitual abortion. Studies by Gomez-Arango et al. found that *Odoribacter* was negatively correlated with systolic blood pressure at 16 weeks of pregnancy in women with healthy pregnancies (40), suggesting a protective role in maintaining normal blood pressure levels during pregnancy. However, it is noteworthy that *Coprococcus3* has been reported to be positively associated with certain diseases, such as reduced immunity (34, 41, 42), but its role in miscarriage has not yet been reported. In our MR study, we speculate that *Coprococcus3*’s preventive role against miscarriage may stem from its ability to produce butyrate (43, 44). Nonetheless, whether *Coprococcus3* and *Odoribacter* influence the occurrence of habitual miscarriages through specific pathways and their mechanisms of action has not been detailed in clinical studies yet. These microbiota undoubtedly warrant further research.

However, this study has certain limitations. First, our data were primarily drawn from European populations provided by the MiBioGen and FinnGen consortia, which limits the diversity of the population in our MR study. Second, our analysis only explored potential causal relationships between GM at the genus level and miscarriage. Third, our Mendelian Randomization (MR) analysis primarily relied on the significance (*p* < 0.05) of the Inverse Variance Weighted (IVW) method. It is prudent to interpret the significance derived from a single method cautiously. Therefore, future studies should aim to validate these findings with larger datasets and explore other robust MR methods to further strengthen causal inference. Fourth, the MR analysis results did not meet the Bonferroni correction threshold [*p* = 4.20 × 10^-4^ (0.05/119)], meaning the associations in this study are not statistically significant. Hence, these findings are indicative of potential associations rather than definitive evidence. More research is needed to reveal the specific mechanisms involved.

## 5 Conclusion

Overall, this study utilized two-sample MR to explore the potential causal relationships between the GM and miscarriage, identifying both beneficial and harmful microbial groups that affect miscarriage. This research could potentially assist in the early prevention of miscarriage and provide new insights into its treatment.

## Data availability statement

The original contributions presented in the study are included in the article/**Supplementary Material**. Further inquiries can be directed to the corresponding author.

## Ethics statement

The studies involving humans were approved by Finnish Genome Center Ethics Committee. The studies were conducted in accordance with the local legislation and institutional requirements. Written informed consent for participation was not required from the participants or the participants’ legal guardians/next of kin in accordance with the national legislation and institutional requirements.

## Author contributions

HY: Conceptualization, Data curation, Methodology, Software, Writing – original draft, Investigation, Validation, Writing – review & editing. JC: Conceptualization, Data curation, Methodology, Software, Writing – original draft, Formal analysis, Writing – review & editing. YW: Conceptualization, Data curation, Formal analysis, Investigation, Writing – original draft, Writing – review & editing. YL: Methodology, Writing – original draft, Writing – review & editing. QJ: Conceptualization, Funding acquisition, Resources, Supervision, Writing – original draft, Writing – review & editing.

## References

[1] Quenby S Gallos ID Dhillon-Smith RK Podesek M Stephenson MD Fisher J. Miscarriage matters: the epidemiological, physical, psychological, and economic costs of early pregnancy loss. Lancet. (2021) 397:1658–67. doi: 10.1016/S0140-6736(21)00682-6 33915094

[2] Colley E Hamilton S Smith P Morgan NV Coomarasamy A Allen S. Potential genetic causes of miscarriage in euploid pregnancies: a systematic review. Hum Reprod Update. (2019) 25:452–72. doi: 10.1093/humupd/dmz015 31150545

[3] Regan L Rai R. Epidemiology and the medical causes of miscarriage. Baillieres Best Pract Res Clin Obstet Gynaecol. (2000) 14:839–54. doi: 10.1053/beog.2000.0123 11023804

[4] Devall AJ Coomarasamy A. Sporadic pregnancy loss and recurrent miscarriage. Best Pract Res Clin Obstet Gynaecol. (2020) 69:30–9. doi: 10.1016/j.bpobgyn.2020.09.002 32978069

[5] Carp H. A systematic review of dydrogesterone for the treatment of threatened miscarriage. Gynecol Endocrinol. (2012) 28:983–90. doi: 10.3109/09513590.2012.702875 PMC3518297 22794306

[6] Adak A Khan MR. An insight into gut microbiota and its functionalities. Cell Mol Life Sci. (2019) 76:473–93. doi: 10.1007/s00018-018-2943-4 PMC11105460 30317530

[7] Lynch SV Pedersen O. The human intestinal microbiome in health and disease. N Engl J Med. (2016) 375:2369–79. doi: 10.1056/NEJMra1600266 27974040

[8] Takada K Melnikov VG Kobayashi R Komine-Aizawa S Tsuji NM Hayakawa S. Female reproductive tract-organ axes. Front Immunol. (2023) 14:1110001. doi: 10.3389/fimmu.2023.1110001 36798125 PMC9927230

[9] Qi X Yun C Pang Y Qiao J. The impact of the gut microbiota on the reproductive and metabolic endocrine system. Gut Microbes. (2021) 13:1–21. doi: 10.1080/19490976.2021.1894070 PMC7971312 33722164

[10] Donald K Finlay BB. Early-life interactions between the microbiota and immune system: impact on immune system development and atopic disease. Nat Rev Immunol. (2023) 23:735–48. doi: 10.1038/s41577-023-00874-w 37138015

[11] Inversetti A Zambella E Guarano A Dell'Avanzo M Di Simone N. Endometrial microbiota and immune tolerance in pregnancy. Int J Mol Sci. (2023) 24(3):2995. doi: 10.3390/ijms24032995 36769318 PMC9917440

[12] Giannella L Grelloni C Quintili D Fiorelli A Montironi R Alia S. Microbiome changes in pregnancy disorders. Antioxidants (Basel). (2023) 12(2):463. doi: 10.3390/antiox12020463 36830021 PMC9952029

[13] Martin-Gallausiaux C Marinelli L Blottière HM Larraufie P Lapaque N. SCFA: mechanisms and functional importance in the gut. Proc Nutr Soc. (2021) 80:37–49. doi: 10.1017/S0029665120006916 32238208

[14] Shin D Chang SY Bogere P Won K Choi J-Y Choi Y-J. Beneficial roles of probiotics on the modulation of gut microbiota and immune response in pigs. PloS One. (2019) 14:e0220843. doi: 10.1371/journal.pone.0220843 31461453 PMC6713323

[15] Jin M Li D Ji R Liu W Xu X Feng X. Changes in gut microorganism in patients with positive immune antibody-associated recurrent abortion. BioMed Res Int. (2020) 2020:4673250. doi: 10.1155/2020/4673250 33015167 PMC7520699

[16] Sekula P Del Greco MF Pattaro C Köttgen A. Mendelian randomization as an approach to assess causality using observational data. J Am Soc Nephrol. (2016) 27:3253–65. doi: 10.1681/ASN.2016010098 PMC5084898 27486138

[17] Khasawneh LQ Al-Mahayri ZN Ali BR. Mendelian randomization in pharmacogenomics: The unforeseen potentials. BioMed Pharmacother. (2022) 150:112952. doi: 10.1016/j.biopha.2022.112952 35429744

[18] Liu Y Chen H Feng L Zhang J. Interactions between gut microbiota and metabolites modulate cytokine network imbalances in women with unexplained miscarriage. NPJ Biofilms Microbiomes. (2021) 7:24. doi: 10.1038/s41522-021-00199-3 33731680 PMC7969606

[19] VanderWeele TJ Tchetgen Tchetgen EJ Cornelis M Kraft P. Methodological challenges in mendelian randomization. Epidemiology. (2014) 25:427–35. doi: 10.1097/EDE.0000000000000081 PMC3981897 24681576

[20] Liu Y Xu H Zhao Z Dong Y Wang X Niu J. No evidence for a causal link between Helicobacter pylori infection and nonalcoholic fatty liver disease: A bidirectional Mendelian randomization study. Front Microbiol. (2022) 13:1018322. doi: 10.3389/fmicb.2022.1018322 36406444 PMC9669663

[21] Kurilshikov A Medina-Gomez C Bacigalupe R Radjabzadeh D Wang J Demirkan A. Large-scale association analyses identify host factors influencing human gut microbiome composition. Nat Genet. (2021) 53:156–65. doi: 10.1038/s41588-020-00763-1 PMC8515199 33462485

[22] Xia D Wang J Zhao X Shen T Ling L Liang Y. Association between gut microbiota and benign prostatic hyperplasia: a two-sample mendelian randomization study. Front Cell Infect Microbiol. (2023) 13:1248381. doi: 10.3389/fcimb.2023.1248381 37799337 PMC10548216

[23] Chen S Zhou G Han H Jin J Li Z. Causal effects of specific gut microbiota on bone mineral density: a two-sample Mendelian randomization study. Front Endocrinol (Lausanne). (2023) 14:1178831. doi: 10.3389/fendo.2023.1178831 37645419 PMC10461557

[24] Zhong S Yang W Zhang Z Xie Y Pan L Ren J. Association between viral infections and glioma risk: a two-sample bidirectional Mendelian randomization analysis. BMC Med. (2023) 21:487. doi: 10.1186/s12916-023-03142-9 38053181 PMC10698979

[25] Lee CH Cook S Lee JS Han B. Comparison of two meta-analysis methods: inverse-variance-weighted average and weighted sum of Z-scores. Genomics Inform. (2016) 14:173–80. doi: 10.5808/GI.2016.14.4.173 PMC5287121 28154508

[26] Li B Han Y Fu Z Chai Y Guo X Du S. The causal relationship between gut microbiota and lymphoma: a two-sample Mendelian randomization study. Front Immunol. (2024) 15:1397485. doi: 10.3389/fimmu.2024.1397485 38774867 PMC11106390

[27] Jurkovic D Overton C Bender-Atik R. Diagnosis and management of first trimester miscarriage. BMJ. (2013) 346:f3676. doi: 10.1136/bmj.f3676 23783355

[28] Kapp N Lohr PA. Modern methods to induce abortion: Safety, efficacy and choice. Best Pract Res Clin Obstet Gynaecol. (2020) 63:37–44. doi: 10.1016/j.bpobgyn.2019.11.008 32029379

[29] Ghosh J Papadopoulou A Devall AJ Jeffery HC Beeson LE Do V. Methods for managing miscarriage: a network meta-analysis. Cochrane Database Syst Rev. (2021) 6:CD012602. doi: 10.1002/14651858.CD012602.pub2 34061352 PMC8168449

[30] Mouri Mi Hall H Rupp TJ. Threatened abortion, in: StatPearls (2024). Treasure Island (FL): StatPearls Publishing. Available online at: http://www.ncbi.nlm.nih.gov/books/NBK430747/ (Accessed April 10, 2024).

[31] Ballal SA Veiga P Fenn K Michaud M Kim JH Gallini CA. Host lysozyme-mediated lysis of Lactococcus lactis facilitates delivery of colitis-attenuating superoxide dismutase to inflamed colons. Proc Natl Acad Sci U.S.A. (2015) 112:7803–8. doi: 10.1073/pnas.1501897112 PMC4485081 26056274

[32] Bohlul E Hasanlou F Taromchi AH Nadri S. TRAIL-expressing recombinant Lactococcus lactis induces apoptosis in human colon adenocarcinoma SW480 and HCT116 cells. J Appl Microbiol. (2019) 126:1558–67. doi: 10.1111/jam.14237 30815963

[33] González-Sánchez A Reyes-Lagos JJ Peña-Castillo MA Nirmalkar K García-Mena J Pacheco-López G. Vaginal microbiota is stable and mainly dominated by lactobacillus at third trimester of pregnancy and active childbirth: A longitudinal study of ten Mexican women. Curr Microbiol. (2022) 79:230. doi: 10.1007/s00284-022-02918-1 35767085

[34] Liu B Liu Z Jiang T Gu X Yin X Cai Z. Univariable and multivariable Mendelian randomization study identified the key role of gut microbiota in immunotherapeutic toxicity. Eur J Med Res. (2024) 29:161. doi: 10.1186/s40001-024-01741-7 38475836 PMC10929167

[35] Dimitriadis E Menkhorst E Saito S Kutteh WH Brosens JJ. Recurrent pregnancy loss. Nat Rev Dis Primers. (2020) 6:98. doi: 10.1038/s41572-020-00228-z 33303732

[36] Youssef A Vermeulen N Lashley EELO Goddijn M van der Hoorn MLP. Comparison and appraisal of (inter)national recurrent pregnancy loss guidelines. Reprod BioMed Online. (2019) 39:497–503. doi: 10.1016/j.rbmo.2019.04.008 31182358

[37] Di Simone N De Spirito M Di Nicuolo F Tersigni C Castellani R Silano M. Potential new mechanisms of placental damage in celiac disease: anti-transglutaminase antibodies impair human endometrial angiogenesis. Biol Reprod. (2013) 89(4):88. doi: 10.1095/biolreprod.113.109637 23966323

[38] Masucci L D’Ippolito S De Maio F Quaranta G Mazzarella R Bianco DM. Celiac disease predisposition and genital tract microbiota in women affected by recurrent pregnancy loss. Nutrients. (2023) 15:221. doi: 10.3390/nu15010221 36615877 PMC9823693

[39] Tersigni C D’Ippolito S Di Nicuolo F Marana R Valenza V Masciullo V. Recurrent pregnancy loss is associated to leaky gut: a novel pathogenic model of endometrium inflammation? J Transl Med. (2018) 16:102. doi: 10.1186/s12967-018-1482-y 29665864 PMC5905157

[40] Gomez-Arango LF Barrett HL McIntyre HD Callaway LK Morrison M Dekker Nitert M. Increased systolic and diastolic blood pressure is associated with altered gut microbiota composition and butyrate production in early pregnancy. Hypertension. (2016) 68:974–81. doi: 10.1161/HYPERTENSIONAHA.116.07910 27528065

[41] Liu X Qi X Han R Mao T Tian Z. Gut microbiota causally affects cholelithiasis: a two-sample Mendelian randomization study. Front Cell infection Microbiol. (2023) 13:1253447. doi: 10.3389/fcimb.2023.1253447 PMC10591199 37876873

[42] Hu X Binxu Q Shao G-Z Huang Y Qiu W. Gut microbiota, circulating metabolites, and gallstone disease: a Mendelian randomization study. Front Microbiol. (2024) 15:1336673. doi: 10.3389/fmicb.2024.1336673 38333586 PMC10850572

[43] Parada Venegas D de la Fuente MK Landskron G González MJ Quera R Dijkstra G. Short chain fatty acids (SCFAs)-mediated gut epithelial and immune regulation and its relevance for inflammatory bowel diseases. Front Immunol. (2019) 10:277. doi: 10.3389/fimmu.2019.00277 30915065 PMC6421268

[44] Wang J Zhu N Su X Gao Y Yang R. Gut-microbiota-derived metabolites maintain gut and systemic immune homeostasis. Cells. (2023) 12:793. doi: 10.3390/cells12050793 36899929 PMC10000530

