# Supplementary material for: Assessing causal relationships between gut microbiota and abortion: evidence from two sample Mendelian randomization analysis
Source: Front Endocrinol (Lausanne). 2024 Jul 10;15:1415730. doi: 10.3389/fendo.2024.1415730 (PMC11266152; doi:10.3389/fendo.2024.1415730)

Supplementary Material

**Supplementary Figure S1.** Funnel plots for causal effects of specific GM on Spontaneous abortion and Habitual aborter risk with individual SNPs. A-B): Spontaneous abortion; C-E): Habitual aborter.


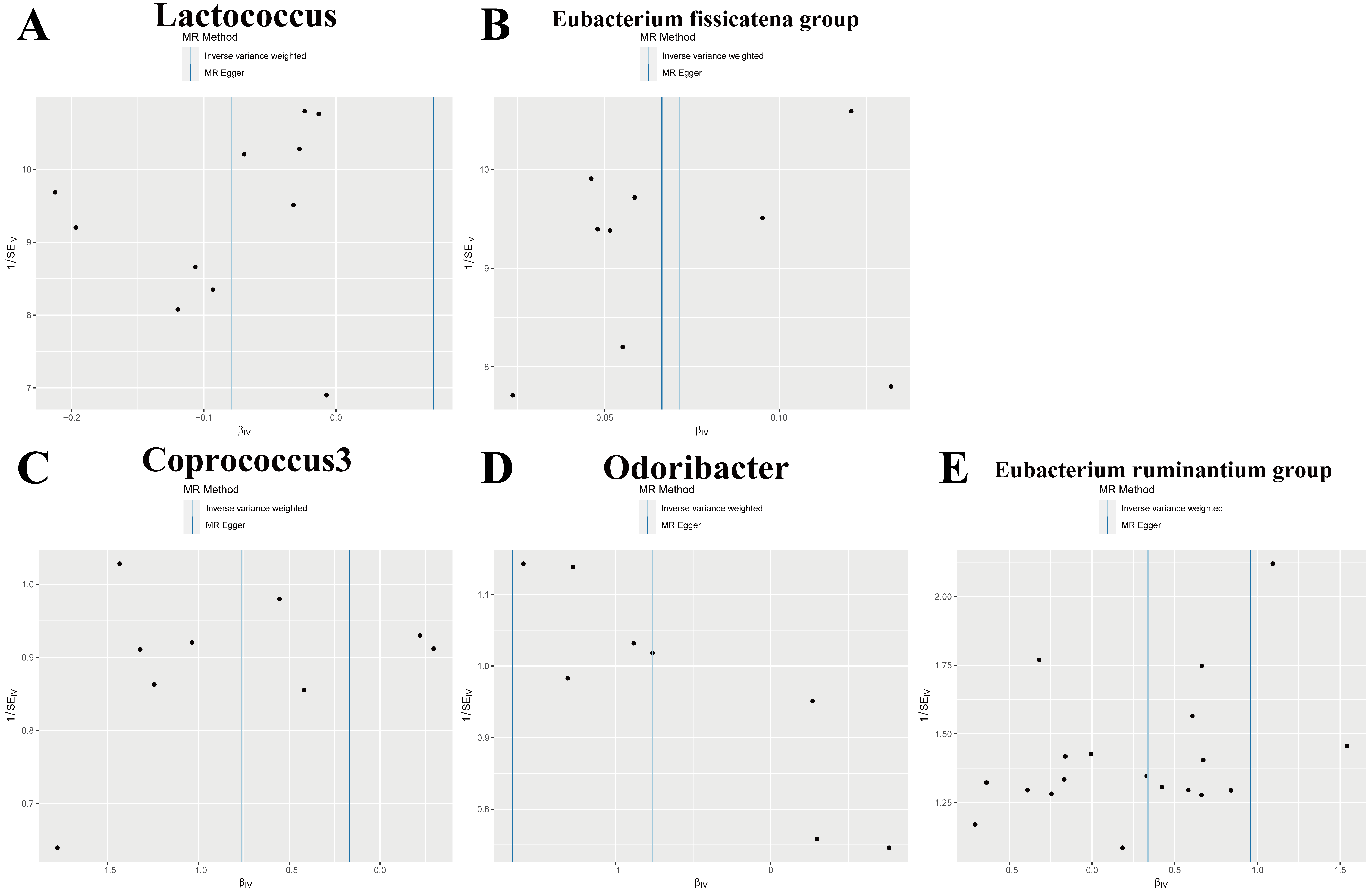


**Supplementary Figure S2.** Forest plots for causal effects of specific GM on Spontaneous abortion and Habitual aborter risk with individual SNPs. A-B): Spontaneous abortion; C-E): Habitual aborter.


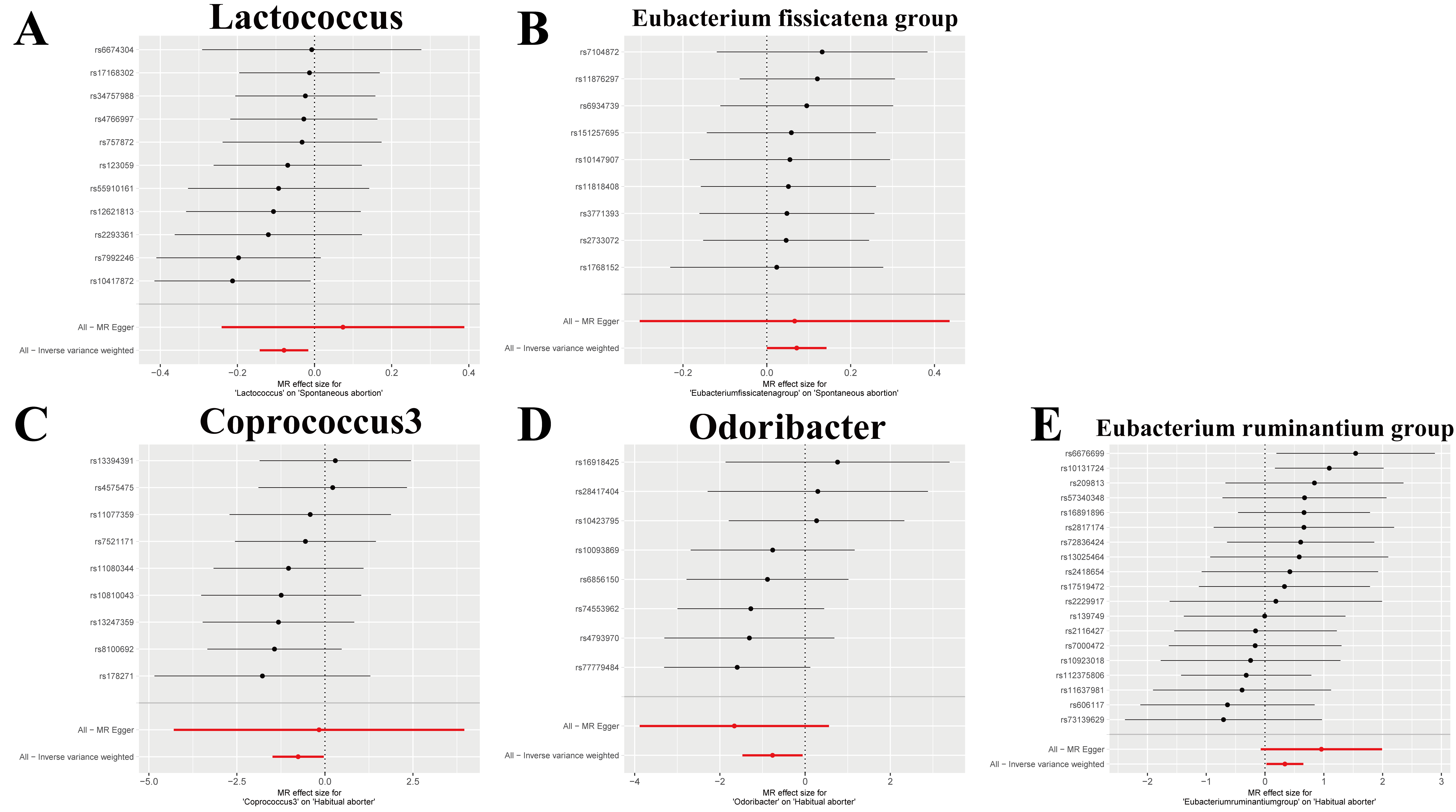

Supplement: Supplementary file 2 [file DataSheet_1.docx]
